# Emerging role of microbes in cancer development and anti-cancer therapy

**DOI:** 10.1016/j.ebiom.2024.105067

**Published:** 2024-03-13

**Authors:** eBioMedicine

Despite remarkable breakthroughs in cancer research, existing anti-cancer treatments still have many challenges, such as high toxicity to healthy cells and rapidly developing drug resistance. In-depth studies of human microbes especially those in the past two decades have not only revealed an intricate interaction between microbes and the development of different types of cancer, but have also provided a promising anti-cancer approach targeting human microbes.

Numerous studies in animals and in people with cancer have shown that microbes have both carcinogenic and therapeutic effects via various mechanisms. In 2011, a paper published in *Proc Natl Acad Sci U S A* showed that *Helicobacter pylori* could induce DNA double-strand breaks in nuclear DNA of gastric epithelial cells. A 2020 study published in *Gut* showed that *Fusobacterium nucleatum* triggered pro-inflammation by activating TLR4 and NF-κB. In addition to direct interaction with host cells, bacterial metabolites can elicit immunosuppressive effects of a host immune system and help cancer cells escape from the immunosurveillance of host cells. In 2022, Yu and colleagues reported in *Gut Microbes* that gut microbes promote pancreatic ductal adenocarcinoma by inhibiting natural-killer cell infiltration and activity. In the same year, a paper by Hezaveh and colleagues published in *Immunity* found that tryptophan metabolites produced by *Lactobacillus* can inhibit intratumoural accumulation of IFNγ^+^CD8^+^ T cells by activating the arylhydrocarbon receptor in macrophages. Aside from cancer-promoting microbes, some normal microbiota are crucial in maintaining a functional host immune system. Thus, dysbiosis of typical gut microbiome increases the risk of cancer occurrence. In their 2023 research paper published in *Nature*, Park and colleagues identified that *Coprobacillus cateniformis* mediates anti-tumour immunity by downregulating the expression of programmed cell death protein ligand 2 on dendritic cells.

An altered microbiome has been related to specific types of cancer and to efficacy of anticancer treatment, suggesting that the microbial signature could be a biomarker for cancer diagnosis or prognosis. In a 2023 prospective, nested case–control study published in *eBioMedicine*, Shi and colleagues showed that circulating markers of microbial translocation, including lipopolysaccharide-binding protein soluble CD14, and endotoxin core antibody IgM, could be used to evaluate the risk of colorectal cancer. In another study published in 2023 in *J Clin Oncol*, the oral-to-gut ratio of *Gammaproteobacteria* was suggested as a non-invasive method for early screening of pancreatic cancer. However, broad validation in larger cohorts of patients, as well as functional studies, will be required before its clinical application.

Increasing research to improve understanding of host–microbe interactions will help identify microbial-based therapies for personalised cancer treatment. The ability of gut microbiota to regulate host immunity warrants further investigation. Griffin and colleagues showed in their 2021 *Science* paper that *Enterococcus* promoted the generation of immune active muropeptides by producing orthologs of NlpC/p60 peptidoglycan hydrolase, which, in turn, increase the efficacy of anti-PD-L1 treatment. Similarly, the 2022 publication by Si and colleagues in *Gut* suggested that oral supplementation of live *Lactobacillus rhamnosus* GG (LGG) could improve the therapeutic efficacy of immune checkpoint blockade agents. Mechanistically, they revealed that LGG stimulated the production of type I interferon in dendritic cells and activated cytotoxic CD8^+^ T cells. In addition to immune checkpoint therapy, gut microbes also have an effect on chimeric antigen receptor T-cell (CAR-T) therapy. In 2022, a retrospective cohort study published in *Nat Med* by Smith and colleagues reported an association between faecal microbial profile and patient response to CD19 CAR-T cell therapy against B-cell lymphoma and leukaemia. Their study indicated the promotion of *Ruminococcus* and *Faecalibacterium* in CD19 CAR-T cell therapy, and *Veillonellaceae* was associated with a decreased clinical response. In a 2023 study published in *eBioMedicine*, Joachim and colleagues established a genetically modified mouse model with interferon alfa receptor 1 deficiency to explore the role of bacterial-derived metabolite desaminotyrosine in immune checkpoint inhibitor therapy. The results showed that oral administration of desaminotyrosine promoted the efficacy of anti-CTLA-4 or anti-PD-1 agents and slowed tumour growth.

The most common microbial-based interventions for cancer therapy include faecal microbial transplantation (FMT), prebiotics or probiotics, and dietary intervention. Each of these approaches has promising potential in either improving efficiency or reducing side-effects of cancer treatment. In a first-in-human, phase 1 clinical trial published in *Science* in 2020, Baruch and colleagues reported that FMT induced CD8+ T cell infiltration and improved immune activity in patients with metastatic melanoma receiving anti-PD-1 immunotherapy. In 2022, a randomised controlled trial published in *Nat Med* by Dizman and colleagues showed that, when combined with nivolumab–ipilimumab treatment, the *Bifidobacterium*-promoting bacterial product CBM588 significantly extended the progression-free survival of patients with metastatic renal cell carcinoma. In a phase 1 clinical trial published in *Nat Med* in 2023, an international team from Canada, France, and the UK conducted a multicentre study to examine the effect of FMT on immunotherapy with nivolumab or pembrolizumab in patients with advanced melanoma. Although the trial was not designed to measure efficacy, longitudinal microbiome profiling showed that 65% of patients who acquired the microbiome of the donor had a clinical response to the combination treatment.

Most previous studies on the human microbiome have been focused on gut microbiota. However, novel discoveries have revealed a functional role for intra-tumour microbiota in regulating cancer development and progression. In a 2020 *Science* article, Nejman and colleagues surveyed bacteria from seven types of human tumours and found a specific bacterial signature associated with each cancer type. Furthermore, in a *Cell* paper published in 2022, Fu and colleagues associated tumour-resident microbiota with cancer metastasis. In a mouse model of spontaneous breast-tumours, the authors showed that reduction of intra-tumour bacteria significantly delayed lung metastasis but did not affect tumour growth. Their findings suggest that circulating tumour cells carrying intra-tumour bacteria might promote host-cancer cell survival by reorganising actin cytoskeleton and enhancing resistance to fluid shear stress.

Epidemiological studies have highlighted an unexplained increased in young-onset (age <50 years) colorectal cancer globally. To explore its pathogenesis, Barot and colleagues compared the tumoural microbial profile of young-onset colorectal cancer with that of average-onset (age >60 years) colorectal cancer. In a study published in *eBioMedicine* in 2024, they reported that patients with young-onset colorectal cancer had a distinct intra-tumoural microbiome, suggesting a unique pathogenesis of young-onset colorectal cancer. More importantly, the dysbiosis signatures of the microbiome were also associated with differences in tumour size, tumour location cancer stage, and clinical outcomes of young-onset and average-onset colorectal cancer. These findings will further update understanding about cancer and cancer therapy.

Despite the potential benefits offered by microbial-based cancer therapy, many challenges need to be overcome before the therapy can be translated into clinical tools. Future clinical trials should focus on large cohorts to assess comorbidities, sampling methods, and confounding among patients. *eBioMedicine*, A Part of The Lancet Discovery Science, publishes important studies on human microbiome in cancer research. We will continue as a publishing platform for translational studies about human diseases, including microbial-based anti-cancer treatments.

